# Characterization of *Anopheles gambiae* D7 salivary proteins as markers of human–mosquito bite contact

**DOI:** 10.1186/s13071-021-05130-5

**Published:** 2022-01-08

**Authors:** Brenda Oseno, Faith Marura, Rodney Ogwang, Martha Muturi, James Njunge, Irene Nkumama, Robert Mwakesi, Kennedy Mwai, Martin K. Rono, Ramadhan Mwakubambanya, Faith Osier, James Tuju

**Affiliations:** 1 KEMRI-Wellcome Trust Research Programme CGMRC, P.O. Box 230-80108, Kilifi, Kenya; 2 Egerton University, P.O. Box 536-20115, Nakuru, Kenya; 3 Pwani University, P.O. Box 195-80108, Kilifi, Kenya; 4 Heidelberg University Hospital, Neuenheimer Feld, 672 69120 Heidelberg, Germany; 5 School of Public Health, University of the Witwatersrand, 1 Jan Smuts Avenue, Braamfontein 2000, Johannesburg, South Africa

**Keywords:** Biomarker of exposure, *Anopheles gambiae*, *Plasmodium falciparum*, Infectious bites, Malaria

## Abstract

**Background:**

Malaria is transmitted when infected *Anopheles* mosquitoes take a blood meal. During this process, the mosquitoes inject a cocktail of bioactive proteins that elicit antibody responses in humans and could be used as biomarkers of exposure to mosquito bites. This study evaluated the utility of IgG responses to members of the *Anopheles gambiae* D7 protein family as serological markers of human–vector contact.

**Methods:**

The D7L2, D7r1, D7r2, D7r3, D7r4 and SG6 salivary proteins from *An. gambiae* were expressed as recombinant antigens in *Escherichia coli*. Antibody responses to the salivary proteins were compared in Europeans with no prior exposure to malaria and lifelong residents of Junju in Kenya and Kitgum in Uganda where the intensity of malaria transmission is moderate and high, respectively. In addition, to evaluate the feasibility of using anti-D7 IgG responses as a tool to evaluate the impact of vector control interventions, we compared responses between individuals using insecticide-treated bednets to those who did not in Junju, Kenya where bednet data were available.

**Results:**

We show that both the long and short forms of the D7 salivary gland antigens elicit a strong antibody response in humans. IgG responses against the D7 antigens reflected the transmission intensities of the three study areas, with the highest to lowest responses observed in Kitgum (northern Uganda), Junju (Kenya) and malaria-naïve Europeans, respectively. Specifically, the long form D7L2 induced an IgG antibody response that increased with age and that was lower in individuals who slept under a bednet, indicating its potential as a serological tool for estimating human–vector contact and monitoring the effectiveness of vector control interventions.

**Conclusions:**

This study reveals that D7L2 salivary antigen has great potential as a biomarker of exposure to mosquito bites and as a tool for assessing the efficacy of vector control strategies such as bednet use.

**Graphical abstract:**

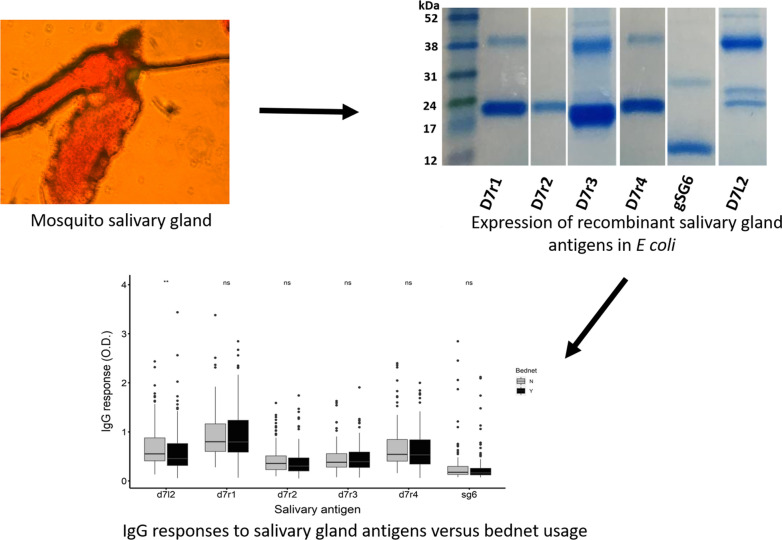

**Supplementary Information:**

The online version contains supplementary material available at 10.1186/s13071-021-05130-5.

## Background

The female *Anopheles* mosquito is haematophagous and requires blood from a vertebrate host to produce eggs. To enable them to feed, mosquitoes have evolved potent bioactive components in their saliva to counteract the vertebrate’s response to tissue injury. The effects of mosquito saliva when injected into the vertebrate host include inhibition of vasoconstriction, platelet aggregation and coagulation to keep blood flowing during the blood meal, and anti-inflammatory and immunomodulatory agents that counteract local immune responses of the host at the vascular probing site [1]. On the other hand, the human host mounts an immune response to the mosquito salivary components to counteract its effects. The levels of anti-mosquito saliva antibodies can possibly reflect the extent of human exposure to mosquito bites at the individual and population levels. Indeed, feasibility studies carried out show that children resident in an area with seasonal malaria transmission developed anti-mosquito saliva immunoglobulin G (IgG) that was correlated with mosquito density [2] and that declined rapidly upon introduction of insecticide-treated bednets [3].

However, given the practical limitations with collection of mosquito saliva for large-scale epidemiological analysis, it is important to identify and validate specific salivary gland antigens that are immunogenic when injected in human subjects. These represent potential markers to measure human–vector exposure by simple immunoassays. These measurements would be helpful in monitoring transmission and the effectiveness of vector control measures. Proof of concept has been demonstrated using the *Anopheles gambiae* salivary gland protein 6 (gSG6), which has proven to be a good marker of exposure to malaria vectors in multiple areas with varying rates of malaria transmission [4–10] and for evaluating the impact of vector control strategies. Specifically, the gSG6-P1 peptide derived from the N-terminal end of this protein has been shown to elicit responses that delineate the level of human–vector exposure with even greater specificity [5].

A limitation to this approach is that not all mosquito bites lead to infection. When sampled in the field, only a fraction of mosquitoes are infectious even in areas with high transmission intensity as measured by the sporozoite rate [11]. As a result, measuring antibody responses to antigens that are constitutively expressed in the salivary glands of mosquitoes may not distinguish infective from non-infective bites and may therefore overestimate malaria transmission metrics. The identification of mosquito salivary gland antigens shown to be upregulated in the salivary gland of *Plasmodium falciparum*-infected mosquitoes may provide a more optimal alternative to precisely estimate malaria transmission metrics by distinguishing between infective and non-infective bites. Marie and colleagues characterized the *An. gambiae* sialome in the presence and absence of *P. falciparum* infection. They identified gSG6 and the long form of D7 among the proteins upregulated by infection, and hence potential candidate markers of human exposure to infective bites [12]. As gSG6 and gSG6-P1 have previously been validated as good biomarkers of exposure, it is plausible that they are potential biomarkers of infective mosquito bites [5, 7, 12–14]. Here, we investigated members of the *An. gambiae* D7 family salivary gland antigens as potential markers of human–vector exposure.

The D7 protein family is among the most abundant salivary gland antigens, and they are expressed only by adult female mosquitoes [15–17]. In *An. gambiae*, D7 forms a multigene family consisting of three long D7 proteins (D7L1, D7L2 and D7L3) and five short D7 forms (D7r1, D7r2, D7r3, D7r4 and D7r5) located on the 3R-30B chromosome [5, 17–19]. Members of this family have been shown to play an important role in blood-feeding by inhibiting platelet aggregation, blood coagulation and inflammation in the vertebrate host [20]. The aim of this study was to explore whether D7 proteins in *An. gambiae* elicit antibody responses in exposed individuals and whether these responses are related to the amount of human–vector exposure at the population level.

## Methods

### Study design and setting

This study used archived plasma samples from a well-established long-term malaria immunological cohort in Kilifi South in the coastal area of Kenya (Additional file 1: Fig. S1). Briefly, plasma was obtained from children in a cross-sectional bleed carried out in April 2014, at the beginning of the malaria season. Parasite prevalence at the cross-sectional bleed was 13.4%. The children were then followed up on a weekly basis at their home for any malaria cases for 1 year. Suspected cases were screened by rapid diagnostic test in the field and confirmed by microscopy. In addition, cases of malaria reported between the weekly visits were diagnosed and treated at a local dispensary. We analysed plasma samples collected during the April 2014 cross-sectional survey (*n* = 299) for participants aged 1–14 years. A survey was carried out at the end of the cross-sectional bleed to establish the penetration and use of bednets among the study participants during the study period. There has been a temporal shift from *An. gambiae* sensu stricto (s.s.) and *An. fenestus* to an increase in *An. arabiensis* as the main vector population in this study area [21].

In addition, to explore the serology of mosquito salivary gland proteins from a high-transmission setting, we assayed plasma samples collected from healthy community children as part of a case–control study evaluating the etiology and pathogenesis of nodding syndrome in the districts of Kitgum in northern Uganda. Sample collection for this study occurred in 2017. For the present analysis, 150 samples were available of 154 recruited in the parent study. This area is located in northern Uganda bordering South Sudan and is inhabited by the Acholi people [22]. The primary economic activity is subsistence farming complemented with small-scale animal husbandry. In this region, malaria transmission occurs all year-round, with a peak following the onset of the rainy season. Further, the region reported a malaria epidemic between 2015 and 2017 [23]. This resurgence was possibly precipitated by halting indoor residual spraying (IRS) campaigns by the national control programme due to a perceived reduction in malaria transmission [24]. Parasite prevalence at the cross-sectional bleed was 55.1%. The entomological inoculation rate (EIR) in Kitgum has not been calculated before but is anticipated to be high. A previous study involving neighbouring Arua and Apac reported average annual EIRs of between 397 and 1586 bites/person, respectively, with *An. gambiae* s.s. as the main vector [25].

### Protein expression and purification

*Anopheles gambiae* Kilifi strain mosquitoes were reared at 27 ± 1 °C, 75 ± 5% relative humidity and 12 h light/dark. The mosquitoes were fed 10% glucose. RNA was extracted from a pool of 5-day-old female *An. gambiae* Kilifi strain mosquitoes (generation 64, *n* = 15) using TRIzol® Reagent (Thermo Fisher Scientific, San Jose, CA, USA). Thereafter, complementary DNA (cDNA) was synthesized using Oligo(dT)_12–18_ and SuperScript II Reverse Transcriptase (Invitrogen, San Diego, CA, USA) according to the manufacturer’s instructions. The D7 family proteins and SG6 were amplified using Go Taq^®^ Hot Start Green Master Mix (Promega, Madison, WI, USA) using the appropriate gene-specific primers (Additional file 2: Table S1) under the following PCR conditions: initial denaturation at 94 °C for 3 min, 40 cycles of 94 °C for 15 s, 55 °C for 30 s and 72 °C for 30 s, and a final elongation of 72 °C for 30 s. The amplified genes were cloned into an expression vector (pEXP-5-CT/TOPO) and transformed into competent *E. coli* BL21 (DE3) strain for protein expression overnight at 37 °C in Auto Induction Media (AIM; Formedium™, Norfolk, UK). The Ni-NTA Purification System under denaturing conditions (Invitrogen, Carlsbad, CA, USA) was used to purify expressed protein following extraction using BugBuster^®^ Protein Extraction Reagent (Novagen, Billerica, MA, USA) and Benzonase Nuclease (Novagen, Billerica, MA, USA). Protein concentration was determined using a Pierce BCA Protein Assay Kit (Thermo Fisher Scientific, San Jose, CA, USA). Protein purity was confirmed using sodium dodecyl sulfate–polyacrylamide gel electrophoresis (SDS-PAGE), and the identity of the proteins was confirmed by mass spectrometry (Additional file 3: Text S1 and Additional file 4: Table S2).

### Enzyme-linked immunosorbent assays (ELISA)

Immune responses against our panel of salivary gland proteins were determined in plasma using a previously described indirect ELISA protocol [26]. Briefly, recombinant antigens were diluted in carbonate-bicarbonate buffer (pH 9.6; Sigma-Aldrich, Darmstadt, Germany) and plated onto Nunc MaxiSorp microtiter plates (Thermo Scientific, Denmark) at 4 °C overnight at a coating concentration of 50 ng/uL. The following day the plate was washed three times with wash buffer (phosphate-buffered saline [PBS], 0.05% Tween 20; Sigma-Aldrich, Darmstadt, Germany) and blocked for 5 h in 1% dried skimmed milk. Samples and standards were diluted 1:1000 in the blocking buffer, added to the plates and incubated at 4 °C overnight. The plates were subsequently washed four times with wash buffer. Detection antibody (horseradish peroxidase [HRP]-conjugated polyclonal rabbit anti-human IgG; Agilent/Dako, Santa Clara, CA, USA) was added to the plates at a dilution of 1:3000 and incubated for 3 h at room temperature. The plates were washed seven times in wash buffer before adding the substrate 3 o-phenylenediamine dihydrochloride (OPD) substrate (Sigma-Aldrich, St. Louis, MO, USA), followed by incubation for 40 min in the dark for colour development. The reaction was stopped using 2 M sulphuric acid and the optical density (OD) read at 492 nm using a BioTek Synergy 4 reader (BioTek Instruments, Winooski, VT, USA).

### Statistical analysis

All data were analysed using R version 3.5.1 (Vienna, Austria). Differences between two paired groups and among several groups were assessed using Wilcoxon rank-sum tests and Kruskal–Wallis test, respectively, with a non-normality distribution assumption. A malaria case was defined as fever (auxiliary temperature ≥ 37.5 °C) plus any parasitaemia for children < 1 year old or fever plus a parasite density ≥ 2500 parasites/µl for children > 1 year old, while in the Ugandan study, malaria status was determined using histidine-rich protein-2 (HRP2) rapid diagnostic tests (CareStart).

## Results

### Protein expression and purification

**Fig. 1:**
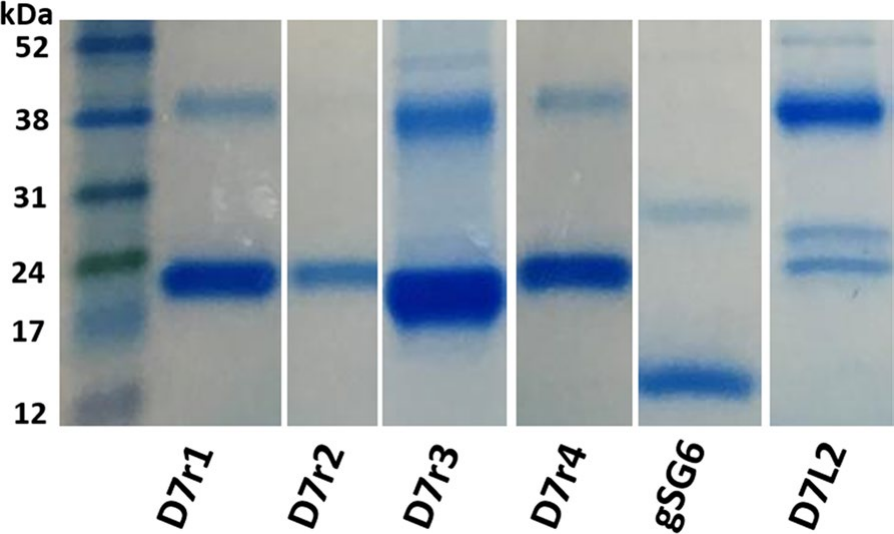
SDS-PAGE image of the recombinant mosquito salivary antigens

The purity of the antigens was evaluated by SDS-PAGE. The predicted sizes of the recombinant mosquito salivary antigens were as follows: D7r1 (18 kDa), D7r2 (18 kDa), D7r3 ( 18 kDa), D7r4 (19 kDa), gSG6 (13 kDa) and D7L2 (36 kDa), corresponding to the protein bands observed after purification (Fig. 1). We however observed additional higher-molecular-weight proteins co-purified with the antigens. We hypothesize that these could be oligomerization products of the purified antigen or endogenous histidine-rich proteins in *E. coli* that have been shown to bind with high affinity to Ni+ affinity purification columns. We however confirmed the identity of the purified products by mass spectrometry (Additional file 4: Table S2).

### Serological responses to mosquito salivary proteins (D7 family and SG6) as markers of exposure

**Fig. 2:**
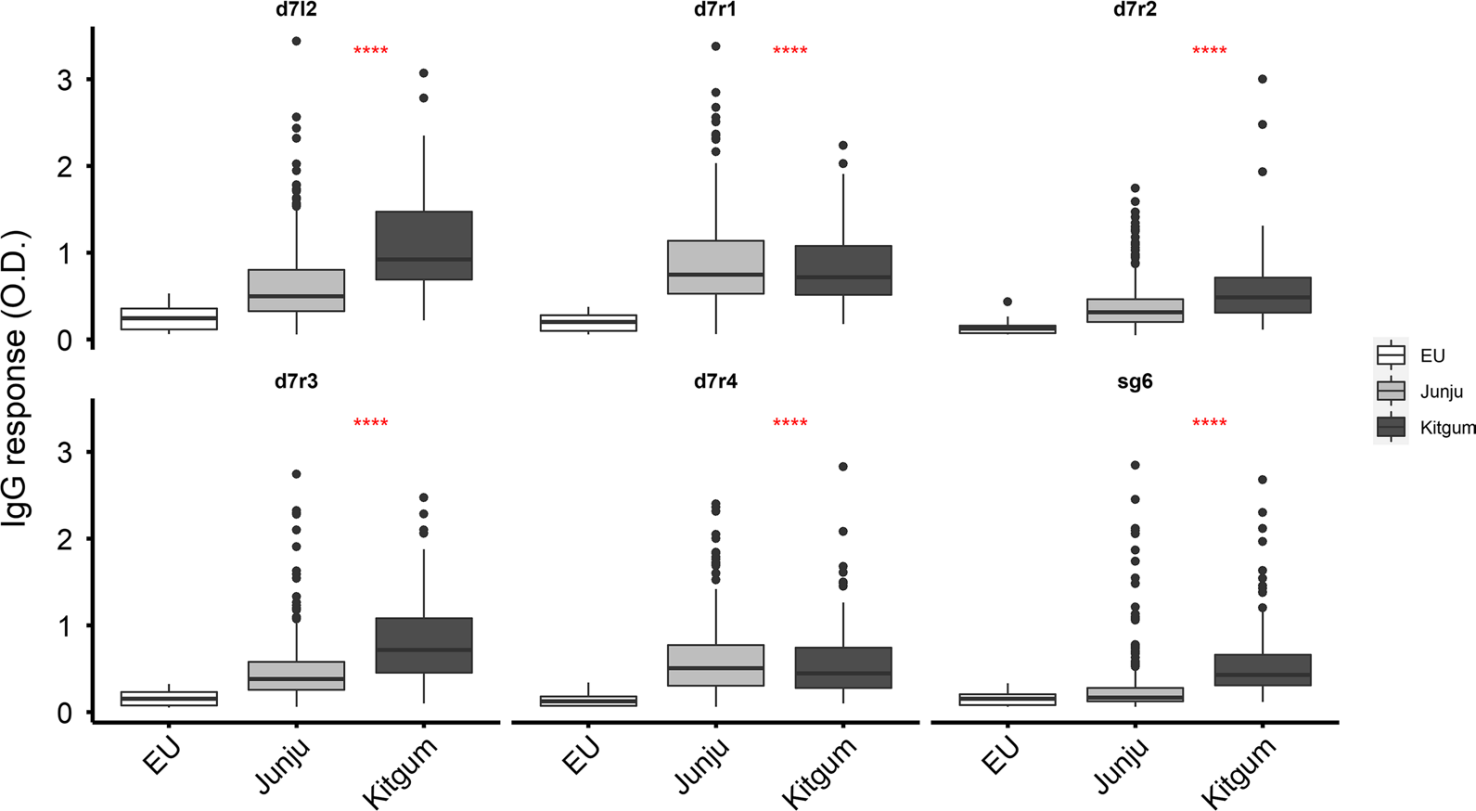
IgG responses to gSG6 and D7 salivary antigens. Plasma IgG levels to D7 proteins were compared between malaria-naïve European adults (*n* = 35), a cohort of children from Junju in Kilifi (*n* = 313) and a cohort Kitgum in northern Uganda (*n* = 147). The *P*-value was determined by the Kruskal–Wallis test (***P < 0.001)

**Fig. 3:**
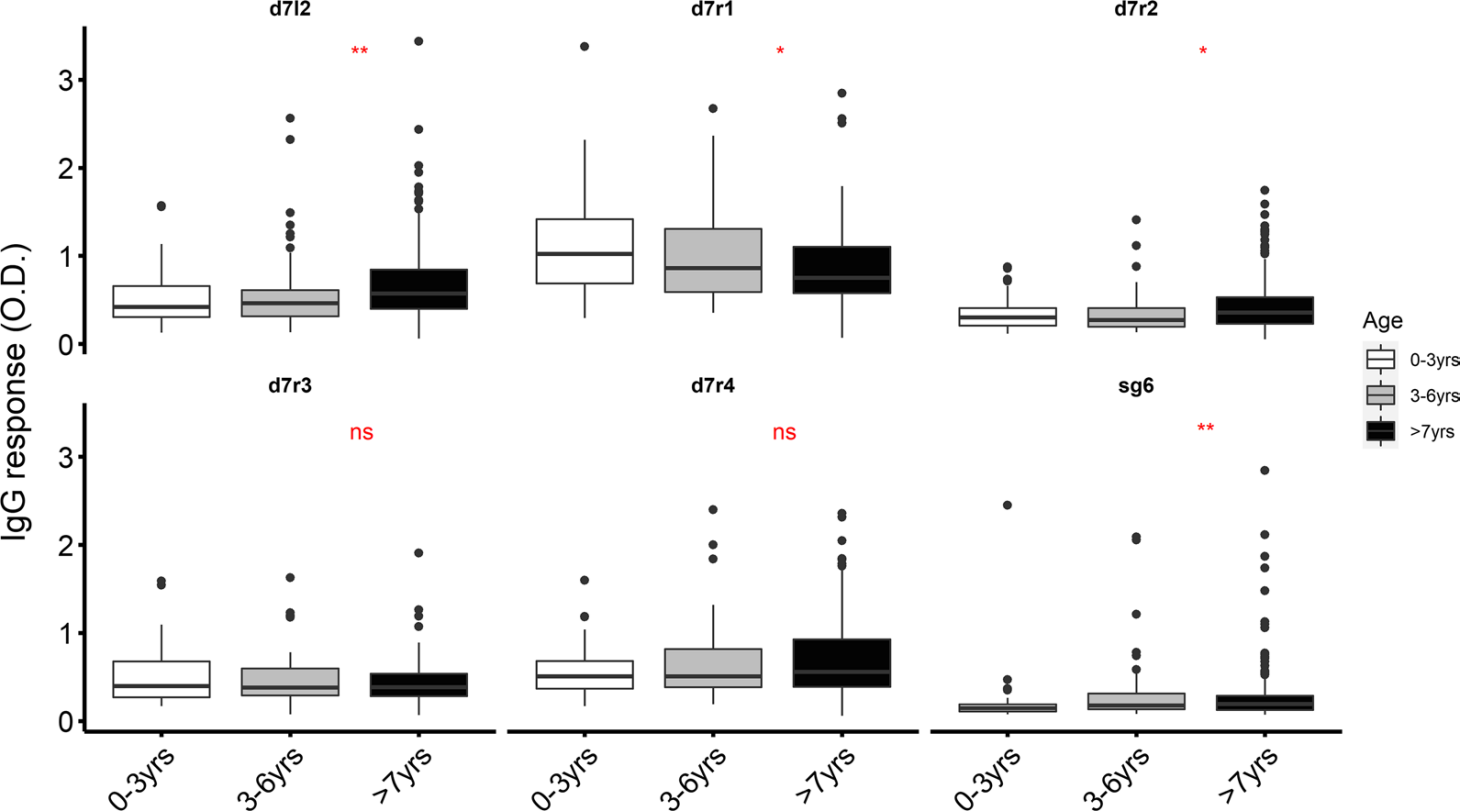
IgG responses to gSG6 and D7 salivary antigens stratified by age among individuals resident in Kilifi, Kenya (0–3 years [*n* = 62], 3–6 years [*n* = 85], > 7 years [*n* = 166]). The *P*-values were determined by the Kruskal–Wallis test (ns, not significant; **P < 0.01; *P < 0.05)

To evaluate immune responses to the family of D7 proteins and SG6, we assayed plasma from malaria-naïve individuals (European individuals with no history of malaria or travel to a malaria-endemic area [*n* = 35]) and malaria-exposed individuals from two independent sites of differing transmission intensity (Junju in coastal Kenya, a region of moderate malaria transmission [*n* = 299], and Kitgum district in northern Uganda (Additional file 1: Fig. S1), a region of high malaria transmission [*n* = 150]). IgG responses to both the short and long forms of D7 proteins as well as gSG6 were significantly higher in malaria-exposed than malaria-naïve European adults (Fig. 2, Additional file 5: Table S3). In addition, IgG responses were significantly higher in samples from northern Uganda than in those from Junju for three of the antigens tested (D7r2, D7r3, D7l2, gSG6), reflecting the difference in transmission intensities between the two study areas (Additional file 6: Table S4). While the responses were generally higher in the exposed individuals, we observed considerable variation within this population, which may be attributable to heterogeneity in exposure caused by climatic and/or socio-economic factors. Further, as age is a reasonable proxy of exposure among lifelong residents of malaria-endemic areas, we stratified the responses by age to explore whether this variation correlated with exposure. First, in the children from Junju, IgG responses to D7L2, D7r2 and SG6 increased significantly with age while those of D7r1 declined (Fig. 3). However, among children from Kitgum, northern Uganda, where malaria transmission is high and the sampled children were much older, there was no difference observed between age groups (Additional file 7: Fig. S2). The high level of transmission in the Ugandan study may result in individuals rapidly acquiring and maintaining saturation levels of antibodies to these antigens at an early age.

### Utility of serological responses to mosquito salivary antigens as a tool for monitoring vector-based control interventions

**Fig. 4:**
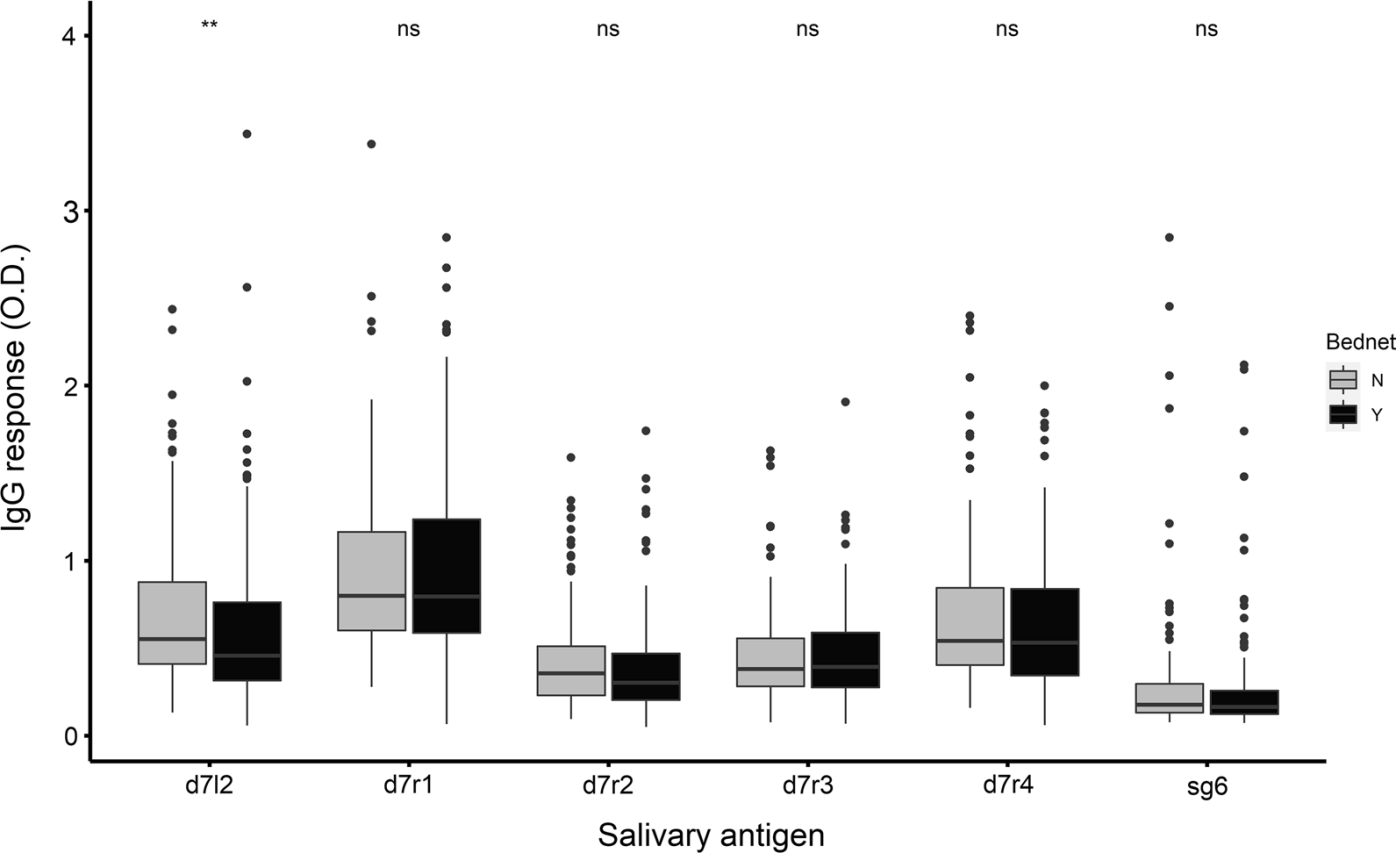
IgG responses to recombinant D7L2 compared between individuals who used insecticide-treated bednets (*n* = 158) and those who did not (*n* = 155). The *P*-value was determined by the Wilcoxon rank-sum test (ns, not significant; ** *P* < 0.01)

We hypothesized that responses to salivary gland antigens may be a useful measure for evaluating the effectiveness of vector-based malaria interventions. To explore this, we compared IgG responses to the recombinant salivary antigens in relation to bednet use among individuals from Junju. Strikingly, we observed significantly lower antibody responses to D7L2 protein in individuals who owned and slept under a bednet as compared with those who did not (Fig. 4). There were no significant differences observed for the other antigens.

## Discussion

Salivary gland antigens are known to play a role in *Plasmodium* transmission and infectivity [27]. They have further been evaluated as markers of human exposure to the mosquito vector [4, 28, 29]. Here, we aimed to evaluate whether D7 proteins in *An. gambiae* elicit antibody responses and whether these responses are related to the level of human–vector exposure. We demonstrate that individuals who are exposed to mosquitoes develop IgG antibodies to members of the D7 protein family. Of interest, when compared with gSG6, responses to the D7 antigens were generally higher, possibly due to the high relative abundance of the latter antigens in the salivary glands of mosquitoes. An analysis of immune responses to peptide fragments spanning the length of the long form of D7 (D7L1) also revealed that this protein has high antigenicity compared with other salivary gland antigens[12].

Importantly, the levels of anti-D7 and anti-gSG6 antibodies reflected differences in transmission intensities at the population level in two rural malaria-endemic areas. We however noted considerable variation in levels of antibodies to the salivary gland antigens among the exposed individuals, possibly reflecting micro-heterogeneity in exposure among residents within the same area. This could therefore be useful as a serological tool to identify hotspots of transmission for a more targeted approach in integrated vector management, particularly in low-transmission settings. It is also plausible that the variation in antibody levels observed is due to inter-individual heterogeneity in the immune response independent of exposure. As we did not have individual-specific metrics of transmission, we were unable to explore this further. We however noted that antibody levels to D7L2, D7r2 and SG6 were higher in the older children, probably reflecting the higher cumulative exposure as individuals have increased vector contact as they grow older [30]. Taken together, these findings imply that the D7 family of antigens is immunogenic in humans and may be a surrogate biomarker of mosquito exposure at the population level.

In previous research, immune responses against mosquito salivary antigens have been measured to evaluate the efficacy of a vector control strategy. A study carried out in Dakar demonstrated that the effective use of insecticide-treated bednets led to a decrease in anti-gSG6-P1 peptide IgG responses [13]. In this study we did not see such differences in the responses to gSG6. This may be due to differences in the antigen used in the assay. The full-length gSG6 recombinant protein used in our study has been shown to elicit high background in individuals with no exposure to mosquito bites, and this might obscure the responses measured by the N-terminal gSG6-P1 peptide which has been shown to have higher specificity [5]. In this study, however, individuals who frequently used insecticide-treated bednets had a significantly lower IgG response to recombinant D7L2 as compared with those who did not, highlighting its utility in evaluating the efficacy of vector control strategies.

As the long form of D7 proteins has been shown to be upregulated in the salivary glands of both *P. falciparum*-infected *An. gambiae* and *Plasmodium berghei*-infected *Anopheles coluzzii* mosquitoes [12], immune responses to D7L2 proteins have further potential to distinguish infectious from non-infectious mosquito bites. This would be particularly helpful in longitudinal prospective cohort studies that evaluate the correlates of naturally acquired immunity. A key limitation of these studies has been the inability to differentiate between truly immune individuals from those who appear immune because they were not exposed to infectious mosquito bites during the follow-up [31], especially in low-transmission settings. This study is an initial step at characterizing the D7 family of antigens as biomarkers of exposure. We however lacked entomological data on exposure. The ability of D7L2 to distinguish infectious from non-infectious mosquito bites will need validation in well-controlled studies where exposure to infected mosquitoes is precisely tracked in a human cohort or suitable animal model. As *Plasmodium* infection upregulates the expression of gSG6 and D7L2 by approximately 1.8- and 1.4-fold respectively, this will also clarify whether such levels of fold-overexpression can elicit a threshold of antibody response that sensitively distinguishes infectious bites. In addition, information on whether the IgG responses are short-lived will be a key factor in their utility as markers of exposure. Ideally, the response should be short enough to detect close temporal changes in malaria transmission such as between the wet and dry seasons. This will also be useful in monitoring the short-term impacts of vector control programmes.

The D7 protein family is widespread among blood-feeding Diptera including anopheline and culicine mosquitoes as well as sand flies [32]. Of interest, the long form of D7 protein has orthologues in *Anopheles funestus*, *Aedes aegypti*, *Culex quinquefasciatus*, *Glossina morsitans* and *Phlebotomus papatasi* with varying pairwise sequence identities (75–27%) [12, 32]. It will be important to explore the extent of cross-reactive responses to the shared orthologues. The specificity of the D7L2 can be further optimized using carefully designed peptides. The feasibility of using D7L2 peptides to detect antibody responses in exposed individuals has already been demonstrated [12].

## Conclusion

Malaria is a major health problem in sub-Saharan Africa. Accurate estimation of the risk of malaria transmission is crucial in employing and monitoring the right tools for the control, management and potential elimination of the disease. This study has shown that D7L2 is a potential biomarker of exposure to anopheline bites and hence a valuable addition to serological tools for estimating human–vector contact. Further studies are needed to precisely establish its role as a marker of infective mosquito bites.

## Supplementary Information

**Additional file 1: Figure S1**. Map of East Africa showing the study areas.

**Additional file 2: Table S1.** Sequences of gene-specific primers used for PCR amplification.

**Additional file 3: Text S1.** Sample preparation, liquid chromatography-tandem mass spectrometry (LC–MS/MS) analysis and antigen validation.

**Additional file 4: Table S2**. Identification of the recombinant mosquito salivary antigens by mass spectrometry.

**Additional file 5: Table S3.** Kruskal–Wallis test comparing differences in responses to salivary gland antigens between the sites.

**Additional file 6: Table S4.** Dunn test post hoc pairwise multiple comparisons for differences in responses to salivary gland antigens between the sites.

**Additional file 7: Figure S2**. IgG responses to gSG6 and D7 salivary antigens stratified by age among children from Kitgum, northern Uganda (06–09 [*n* = 3], 10–12 [*n* = 16], 13–15 [*n* = 90], 16–18 [*n* = 38]).

## Data Availability

The datasets analysed in the current study are available from the corresponding author on reasonable request.

## Abbreviations

ELISA: Enzyme-linked immunosorbent assay
IgG: Immunoglobulin G
LC–MS/MS: Liquid chromatography-tandem mass spectrometry
PBS: Phosphate-buffered saline
PCR: Polymerase chain reaction
