# Supplementary figures and images for: Characterization of Anopheles gambiae D7 salivary proteins as markers of human–mosquito bite contact
Source: Parasit Vectors. 2022 Jan 8;15:11. doi: 10.1186/s13071-021-05130-5 (PMC8742437; doi:10.1186/s13071-021-05130-5)

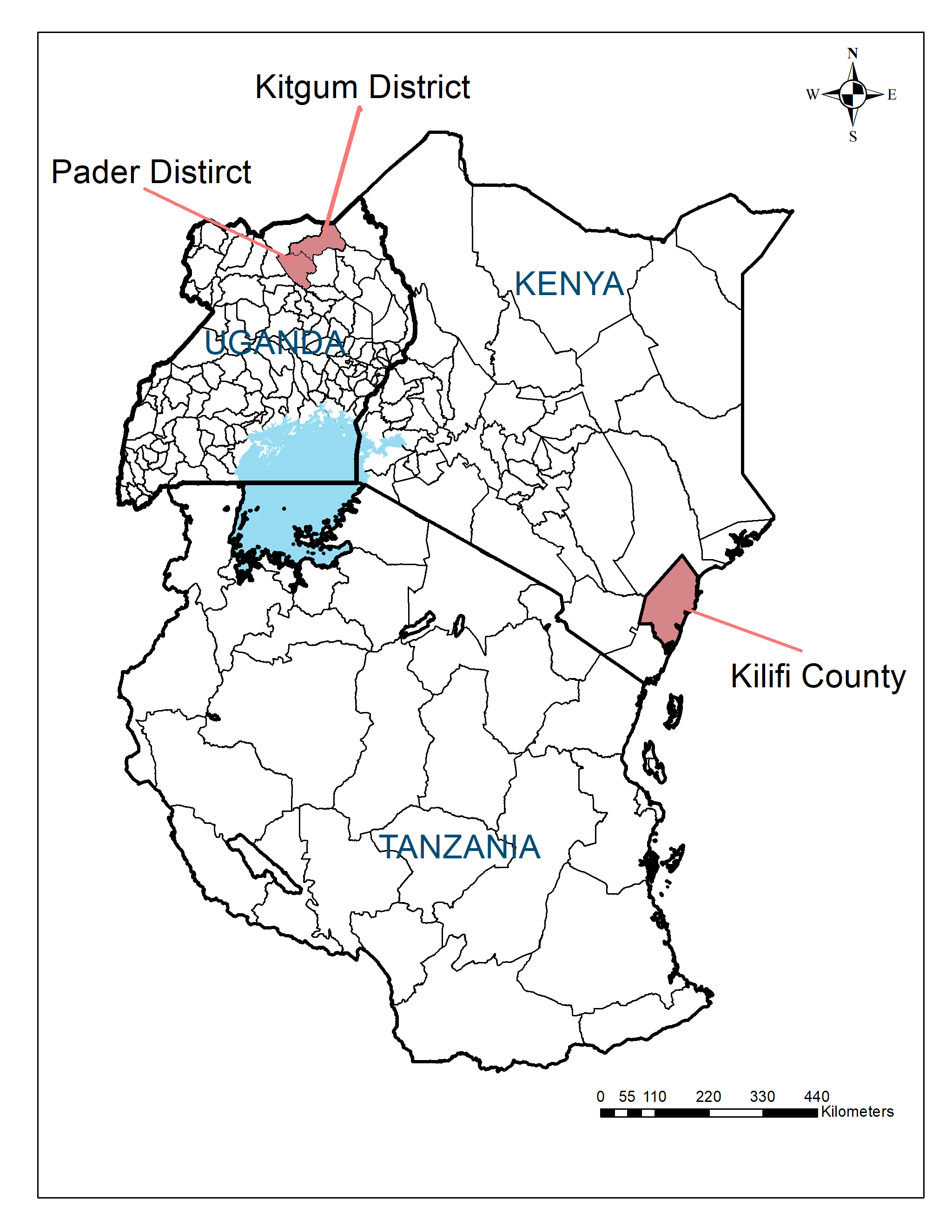

Supplement: Supplementary file 1 — Additional file 1: Figure S1. Map of East Africa showing the study areas. [file 13071_2021_5130_MOESM1_ESM.tif]

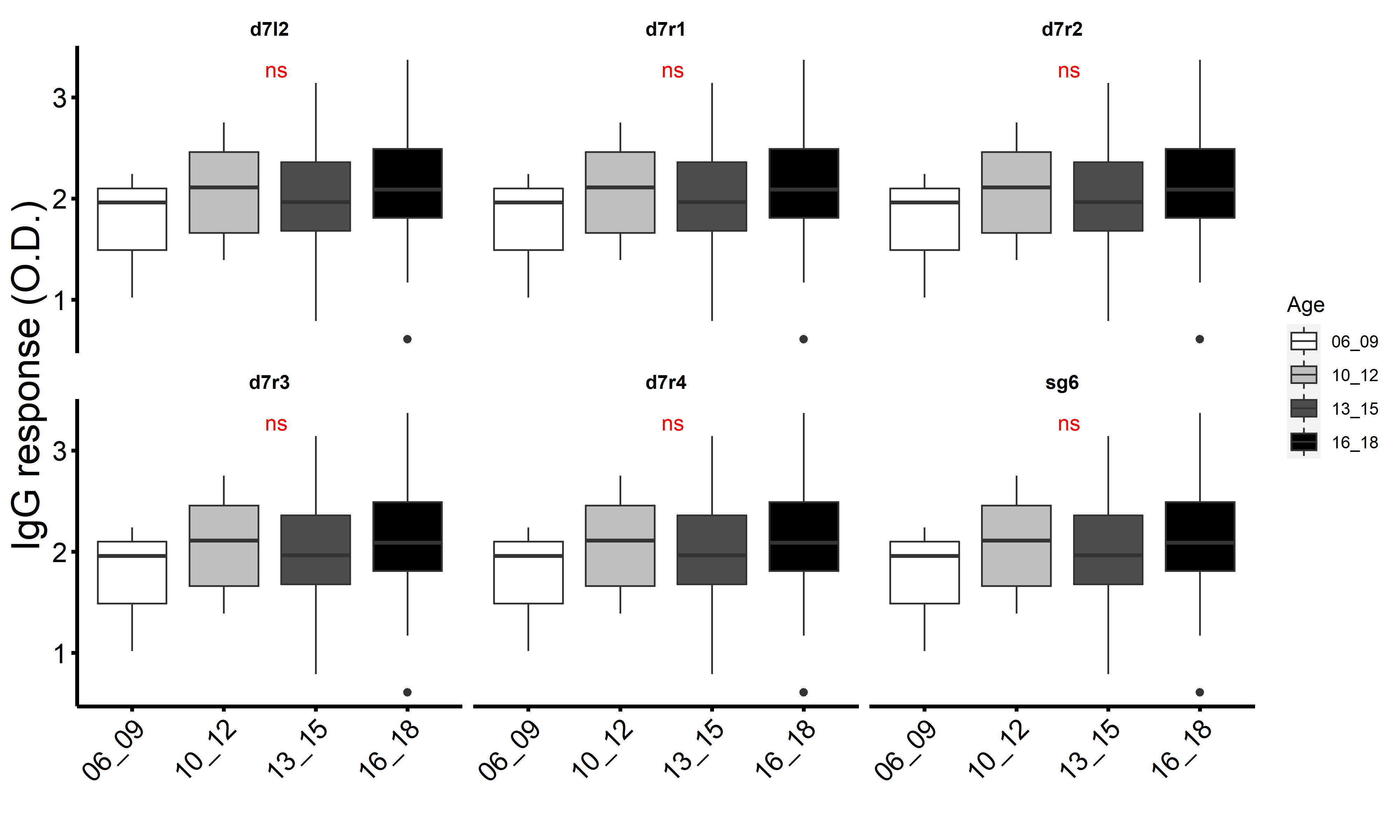

Supplement: Supplementary file 7 — Additional file 7: Figure S2. IgG responses to gSG6 and D7 salivary antigens stratified by age among children from Kitgum, northern Uganda (06–09 [n = 3], 10–12 [n = 16], 13–15 [n = 90], 16–18 [n = 38]). [file 13071_2021_5130_MOESM7_ESM.tif]
