# Supplementary material for: Characterization of Anopheles gambiae D7 salivary proteins as markers of human–mosquito bite contact
Source: Parasit Vectors. 2022 Jan 8;15:11. doi: 10.1186/s13071-021-05130-5 (PMC8742437; doi:10.1186/s13071-021-05130-5)
Supplement: Supplementary file 2 — Additional file 2: Table S1. Sequences of gene-specific primers used for PCR amplification. [file 13071_2021_5130_MOESM2_ESM.docx]

**Table S1.** Sequences of gene specific primers used for PCR amplification.

| Mosquito salivary gland gene | Forward primer sequence  (5ˈ 3ˈ) | Reverse primer sequence  (5ˈ 3ˈ) |
| --- | --- | --- |
| D7L2 | ATGGCTAGCGACATCAACTCGAAATGGCG | GGATCCGAAATGGACAGTTGTTTAGATG |
| D7r1 | ATGGCTAGCAACACGGTTAAGAAGTGTGAGA | GGATCCGTTGCAAATCTTGTCATCGA |
| D7r2 | ATGGCTAGCCGAAAGGAGTCAACGGTGGA | GGATCCGCACAAACCATCATCGATTTCCT |
| D7r3 | ATGGCTAGCAGACAAGAGGAAACGGTTGAAGA | GGATCCGTTACACAGCCCATCATCAA |
| D7r4 | ATGGCTAGCGAGACTGTGCAAGATTGTGAGA | GGATCCGCAGTTTAATGCCTTATCATAATCCT |
| SG6 | GAAAAGGTGTGGGTCGA | CTGCTCCAGGAAGGCCT |
