# Supplementary material for: Characterization of Anopheles gambiae D7 salivary proteins as markers of human–mosquito bite contact
Source: Parasit Vectors. 2022 Jan 8;15:11. doi: 10.1186/s13071-021-05130-5 (PMC8742437; doi:10.1186/s13071-021-05130-5)
Supplement: Supplementary file 3 — Additional file 3: Text S1. Sample preparation, liquid chromatography-tandem mass spectrometry (LC–MS/MS) analysis and antigen validation. [file 13071_2021_5130_MOESM3_ESM.docx]

**Additional file 2:** Text S1**.** SDS-PAGE; Sample preparation, LC-MS/MS analysis, and antigen validation.

1. **SDS-PAGE**

Recombinant protein was mixed with equal volume of 2X Tris-Tricine sample buffer containing 2%w/v β-mercaptoethanol and boiled for 5 minutes using Thermomixer compact heating block (Eppendorf, Hamburg, Germany). The denatured proteins were then loaded onto 16 % Tris-Tricine polyacrylamide gel and separated for 120 minutes at 110 volts using Mini PROTEAN® Tetra Electrophoresis System (Biorad, Hercules, CA, USA). Following electrophoresis, the gels were stained with InstantBlue (Expedeon) and destained in distilled water overnight. To estimate the molecular weights of the protein, comparison was done with Full-Range Rainbow Molecular Weight Marker (GE Healthcare Life Sciences, Malborough, MA, USA).

1. **Sample preparation, LC-MS/MS analysis, and antigen validation.**

To confirm the identity of the proteins, Liquid Chromatography-tandem mass spectrometry (LC-MS/MS) was carried out on the purified proteins. 20 µg/µl of each purified recombinant protein was denatured in 50Mm Tris-HCL(Sigma) containing 8 M urea (Sigma). Proteins were reduced with 40 mM Dithiothreitol (Sigma) at room temperature with shaking for 1 hour (h) and subsequently alkylated in the dark for 1 h with 80 mM iodoacetamide (Sigma). Proteins were precipitated with four times the sample volume of cold acetone (-20°C), for 1h at -20°C and the protein pellet obtained after discarding the supernatant following centrifugation for 10 min at 14,000g at room temperature. Proteins were resuspended in 15µl of 6M urea in 50mM Tis-HCL (pH 8) buffer and digested with trypsin/Lys-C mix (Promega) according to the manufacturer’s instructions using the two step in-solution digestion. Peptides obtained were desalted using P10 C18 pipette ZipTips (Millipore) according to manufacturer’s instructions. Eluted peptides were dried in a Speedvac concentrator (Thermo Scientific) and re-suspended in 15μl resuspension solvent (99% H_2_O, 1% acetonitrile, 0.1% formic acid). Peptides (5μl) were loaded using a Dionex Ultimate 3000 nano-flow ultra-high-pressure liquid chromatography system (Thermo Scientific) on to a 75µm x 2cm C18 trap column (Thermo Scientific). Chromatographic separation of peptides was carried out on a reverse-phase 50cm-long column (Thermo Scientific) maintained at 40°C over a 60-min elution gradient (2 to 40% of mobile phase B; 80% acetonitrile with 0.1% formic acid) at a flow rate of 0.3μl/min. Peptides were measured using LC instrumentation consisting of a Dionex Ultimate 3000 nano-flow ultra-high-pressure liquid chromatography system (Thermo Scientific) coupled via a nano-electrospray ion source (Thermo Scientific) to a Q Exactive Orbitrap mass spectrometer (Thermo Scientific). The ms^1 settings were: Resolution, 70000; Automatic gain control (AGC) target, 3e6; maximum injection time, 100ms; scan range, 380-1600m/z; while the ms^2 settings were: Resolution, 17500; AGC target, 5e4; maximum injection time, 100ms; isolation window, 1.6 m/z. The top 10 most intense ions were selected for ms^2 and fragmented with higher-energy collision fragmentation using normalized collision energy of 28 and these ions were subsequently excluded for the next 20s. Mass spectrometry raw files were searched on Proteome Discoverer software version 1.3.0.339 (Thermo Scientific) using the Mascot server (Matrix Science) using a concatenated database of *Anopheles gambiae* and *Plasmodium berghei* protein FASTA sequences. Cysteine carbamidomethylation was set as a fixed modification and deamidation of asparagine or glutamine and methionine oxidation as variable modifications. The false discovery rate (FDR) was set to 0.01 for both proteins and peptides and a maximum of two missed cleavages were allowed in the database search. A minimum of two unique peptides for a protein were considered a positive identification.
