# Supplementary material for: Characterization of Anopheles gambiae D7 salivary proteins as markers of human–mosquito bite contact
Source: Parasit Vectors. 2022 Jan 8;15:11. doi: 10.1186/s13071-021-05130-5 (PMC8742437; doi:10.1186/s13071-021-05130-5)
Supplement: Supplementary file 4 — Additional file 4: Table S2. Identification of the recombinant mosquito salivary antigens by mass spectrometry. [file 13071_2021_5130_MOESM4_ESM.docx]

**Table S2**. Identification of the recombinant mosquito salivary antigens by mass spectrometry.

| **Target antigen** | **gene ID** | **Theoretical MW (kDa)** | **Antigen(s) identified by Mass Spec** | **# of Ms/Ms unique peptide sequences** | **Sequence coverage** | **Mascot score** |
| --- | --- | --- | --- | --- | --- | --- |
| D7L2 | AGAP008279 | 36.138 | AGAP008279-PA  Long form D7 salivary protein | 19 | 68.67 | 4918.46 |
| D7r1 | AGAP008284 | 18.738 | AGAP008284-PA  hypothetical protein | 1 | 26.51 | 243.70 |
| D7r2 | AGAP008282 | 18.483 | AGAP008282-PA  hypothetical protein | 8 | 61.54 | 9522.16 |
| D7r3 | AGAP008283 | 18.653 | AGAP008283-PA  hypothetical protein | 6 | 56.47 | 4542.68 |
| D7r4 | AGAP008281 | 19.309 | AGAP008281-PA  hypothetical protein | 10 | 63.25 | 4463.76 |
| SG6 | AGAP000150 | 13.091 | AGAP000150-PA  GSG6 salivary protein | 5 | 44.83 | 1673.83 |
