# Supplementary material for: Characterization of Anopheles gambiae D7 salivary proteins as markers of human–mosquito bite contact
Source: Parasit Vectors. 2022 Jan 8;15:11. doi: 10.1186/s13071-021-05130-5 (PMC8742437; doi:10.1186/s13071-021-05130-5)
Supplement: Supplementary file 5 — Additional file 5: Table S3. Kruskal–Wallis test comparing differences in responses to salivary gland antigens between the sites. [file 13071_2021_5130_MOESM5_ESM.docx]

**Table S3.**  Kruskal-Wallis test comparing differences in responses to salivary gland antigens between the sites.

| antigen | .y. | n | statistic | df | p |
| --- | --- | --- | --- | --- | --- |
| d7l2 | OD | 490 | 161.456 | 2 | < 0.0001 |
| d7r1 | OD | 490 | 102.1844 | 2 | < 0.0001 |
| d7r2 | OD | 490 | 106.281 | 2 | < 0.0001 |
| d7r3 | OD | 490 | 137.6841 | 2 | < 0.0001 |
| d7r4 | OD | 490 | 94.40637 | 2 | < 0.0001 |
| sg6 | OD | 490 | 144.2298 | 2 | < 0.0001 |
