# Supplementary material for: Characterization of Anopheles gambiae D7 salivary proteins as markers of human–mosquito bite contact
Source: Parasit Vectors. 2022 Jan 8;15:11. doi: 10.1186/s13071-021-05130-5 (PMC8742437; doi:10.1186/s13071-021-05130-5)
Supplement: Supplementary file 6 — Additional file 6: Table S4. Dunn test post hoc pairwise multiple comparisons for differences in responses to salivary gland antigens between the sites. [file 13071_2021_5130_MOESM6_ESM.docx]

**Table S3.**  DunnTest post hoc pairwise multiple comparisons for differences in responses to salivary gland antigens between the sites.

| antigen | .y. | group1 | group2 | n1 | n2 | statistic | p | p.adj | p.adj.signif |
| --- | --- | --- | --- | --- | --- | --- | --- | --- | --- |
| d7l2 | OD | European | Junju | 35 | 299 | 7.765148 | < 0.0001 | 8.15E-15 | **** |
| d7l2 | OD | European | Kitgum | 35 | 150 | 11.87427 | < 0.0001 | 4.83E-32 | **** |
| d7l2 | OD | Junju | Kitgum | 299 | 150 | 8.45513 | < 0.0001 | 5.58E-17 | **** |
| d7r1 | OD | European | Junju | 35 | 296 | 10.08577 | < 0.0001 | 1.92E-23 | **** |
| d7r1 | OD | European | Kitgum | 35 | 150 | 8.231918 | < 0.0001 | 3.68E-16 | **** |
| d7r1 | OD | Junju | Kitgum | 296 | 150 | -2.56924 | 0.010192 | 0.010192 | * |
| d7r2 | OD | European | Junju | 35 | 297 | 7.696972 | < 0.0001 | 2.79E-14 | **** |
| d7r2 | OD | European | Kitgum | 35 | 150 | 10.14964 | < 0.0001 | 9.98E-24 | **** |
| d7r2 | OD | Junju | Kitgum | 297 | 150 | 5.288301 | < 0.0001 | 1.23E-07 | **** |
| d7r3 | OD | European | Junju | 35 | 299 | 6.687858 | < 0.0001 | 2.26E-11 | **** |
| d7r3 | OD | European | Kitgum | 35 | 150 | 10.74604 | < 0.0001 | 1.86E-26 | **** |
| d7r3 | OD | Junju | Kitgum | 299 | 150 | 8.21981 | < 0.0001 | 4.08E-16 | **** |
| d7r4 | OD | European | Junju | 35 | 279 | 9.68218 | < 0.0001 | 1.08E-21 | **** |
| d7r4 | OD | European | Kitgum | 35 | 150 | 7.787952 | < 0.0001 | 1.36E-14 | **** |
| d7r4 | OD | Junju | Kitgum | 279 | 150 | -2.70891 | 0.00675 | 0.00675 | ** |
| sg6 | OD | European | Junju | 35 | 253 | 2.870643 | 0.004096 | 0.004096 | ** |
| sg6 | OD | European | Kitgum | 35 | 150 | 8.677918 | < 0.0001 | 8.06E-18 | **** |
| sg6 | OD | Junju | Kitgum | 253 | 150 | 10.78411 | < 0.0001 | 1.23E-26 | **** |
